# Development of a Rapid and Sensitive RT-qPCR for the Specific Detection of Citrus Viroid V and Its Field Application

**DOI:** 10.3390/v18030335

**Published:** 2026-03-09

**Authors:** Graziella Agrò, Jesús Ángel Sánchez-Navarro, Vicente Pallás, Grazia Licciardello, Giuseppe Scuderi, Antonino Catara, Andrea Giovanni Caruso, Stefano Panno, Slavica Matić, Salvatore Davino

**Affiliations:** 1 Department of Agricultural, Food and Forest Sciences, University of Palermo, 90128 Palermo, Italy; graziella.agro@unipa.it (G.A.); andreagiovanni.caruso@unipa.it (A.G.C.); stefano.panno@unipa.it (S.P.); 2 Institute of Molecular and Cellular Biology of Plants, Universitat Politecnica de Valencia (UPV)—Consejo Superior de Investigaciones Científicas (CSIC), 46022 Valencia, Spain; jesanche@ibmcp.upv.es (J.Á.S.-N.); vpallas@ibmcp.upv.es (V.P.); 3 Council for Agricultural Research and Economics (CREA), Research Centre for Olive, Fruit and Citrus Crops, 95024 Acireale, Italy; grazia.licciardello@crea.gov.it; 4 Agrobiotech Soc. Coop., Z.I Blocco Palma I, 95121 Catania, Italy; gscuderi@agrobiotech.it (G.S.); antoninocatara@virgilio.it (A.C.)

**Keywords:** CVd-V, RT-qPCR, viroid, citrus

## Abstract

Citrus viroid V (CVd-V), a member of the species *Apscaviroid epsiloncitri* (family *Pospiviroidae*), is a graft-transmitted pathogen that spreads through infected propagation material and has been reported in several countries. Recently, it has been detected for the first time in Italy. Although CVd-V is generally considered asymptomatic, foliar and stem symptoms have been observed in Etrog citron. In several citrus species, mixed infections of CVd-V with other viroids give synergistic effects that cause more severe symptoms. To evaluate its spread, a specific and sensitive RT-qPCR assay was developed. The assay specificity, sensitivity, and performance were compared with a conventional end-point RT-PCR, showing a higher sensitivity and no cross-reactivity with other citrus viroids. Furthermore, the assay was evaluated using two RNA extraction procedures: total RNA extraction with commercial kits and a rapid crude extract preparation using a simple extraction buffer. Moreover, RNA extracts of 111 samples, collected from commercial citrus orchards across the main Sicilian citrus-producing areas and from old varieties maintained in germplasm collection, were analyzed. The RT-qPCR revealed an overall CVd-V incidence of 8.1%. Notably, the crude extract preparation showed a sensitivity comparable with conventional total RNA extraction, with substantial savings in cost and processing time. This finding paves the way for using the developed assay with portable qPCR instruments directly in the field, as well as for routine surveillance analyses in citrus production systems.

## 1. Introduction

The genus *Citrus* (family *Rutaceae*) comprises some of the most important tree crops worldwide, including several fruit-producing species such as oranges, mandarin, grapefruit, lemons, and limes. The Mediterranean citrus industry is flourishing, accounting for over 20% of the global citrus production, including fresh fruit, juice processing, and ornamental purposes [1]. Italy is the second-largest citrus producer in Europe, with production concentrated mainly in Sicily. The total citrus orchard area in Italy covers 164,878 hectares, mainly located in Sicily, in Catania, Siracusa, and Messina provinces [2].

Citrus trees are propagated clonally through grafting, which involves joining scions and rootstocks from different cultivars [3]. The selection of a suitable rootstock–scion combination is crucial for achieving high productivity, mitigating biotic and abiotic stress, and ensuring disease resistance. Sour orange (*Citrus aurantium* L.) was historically the predominant rootstock in the Mediterranean basin [4,5]. However, its use has decreased in recent years due to regulatory restrictions associated with its high susceptibility to *Closterovirus tristezae* (citrus tristeza virus, CTV) [6]. The demand for alternative CTV-tolerant rootstocks has led to the adoption of alternatives, such as hybrids derived from trifoliate orange (*Poncirus trifoliata* L.), but these species have shown increased susceptibility to abiotic stress and biotic diseases, in particular those caused by viroids [7,8].

Viroids are infectious agents consisting of a small, single-stranded circular RNA molecule (246–401 nt). Unlike viruses, they do not possess a capsid protein and lack any protein-coding capacity, relying entirely on the host transcriptional machinery to fulfill their replication cycle [9]. To date, eight viroids have been identified as infectious agents in citrus plants: *Hostuviroid impedihumuli* (hop stunt viroid, HSVd), *Pospiviroid exocortiscitri* (citrus exocortis viroid, CEVd), *Apscaviroid curvifoliumcitri* (citrus bent leaf viroid, CBLVd), *Cocadviroid rimocitri* (citrus bark cracking viroid, CBCVd), *Apscaviroid nanocitri* (citrus dwarfing viroid, CDVd), *Apscaviroid epsiloncitri* (citrus viroid V, CVd-V), *Apscaviroid zetacitri* (citrus viroid VI, CVd-VI), and *Apscaviroid etacitri* (citrus viroid VII, CVd-VII) [10]. Among these, CVd-V (family *Pospiviroidae*) remains the viroid with the most uncertain origin. It was first identified in *Atalantia citroides* L. propagated on rough lemon rootstock and graft-inoculated with an artificial mixture of different viroids [11], despite exhibiting resistance to all previously known citrus viroids [12]. So far, CVd-V has been found in several countries in Africa, Asia, Europe, North America, and Oceania, infecting several different citrus hosts [12,13,14,15,16,17,18]. In California, CVd-V has been classified as a risk-category pathogen, and the use of a certified viroid-free nursery is therefore recommended. According to EU certification regulations, viroid screening is carried out exclusively on original candidate mother plants, but it is only mandatory for HSVd and CEVd.

CVd-V is a 294 nt long ssRNA that adopts the typical rod-shaped secondary structure of members of the *Pospiviroidae* family, which can be functionally separated into five domains containing conserved motifs [11]. Infectivity assays demonstrated that CVd-V induces mild symptoms in *Citrus medica* L. (Etrog citron), including small necrotic lesions and stem cracking. However, when co-infecting with CDVd or CBLVd, CVd-V exhibits a synergistic effect, leading to severe symptoms such as epinasty, midvein lesions, stem cracking, necrotic lesions, and severe stunting [11]. Currently, the symptoms caused by CVd-V alone in commercial varieties are unknown, because commercial trees are frequently infected by multiple viroids [19,20], resulting in complex interactions and symptom expression [21]. Additionally, infected propagation material may remain asymptomatic for years, developing symptoms only upon grafting onto susceptible rootstocks. For these reasons, preventing the spread of CVd-V relies heavily on molecular diagnosis to screen propagation material and ensure viroid-free status. In recent decades, molecular techniques have been widely adopted for CVd-V detection, including reverse transcription–polymerase chain reaction (RT-PCR) [16,17], SYBR green quantitative RT-PCR (RT-qPCR) [15], and, more recently, high-throughput sequencing (HTS) [14].

The presence and distribution of various viroids in Italian citrus cultivars have been evaluated in the Lazio, Toscana, and Campania regions [19,22,23]. However, despite Sicily’s significant contribution to national citrus production, no survey has been conducted in this region. Furthermore, comprehensive studies addressing the distribution of CVd-V in Italy are still lacking, as the viroid was only recently detected in the country [18]. For this reason, the aim of the present study was to develop a sensitive and specific probe-based RT-qPCR for CVd-V detection. In addition, CVd-V occurrence was assessed in Sicilian citrus orchards, including multiple commercial cultivars, old varieties from germplasm collection, and several grafting combinations.

## 2. Materials and Methods

### 2.1. RT-qPCR Primers and Probe Design

A novel primer pair and a TaqMan probe were designed for the specific detection of CVd-V by RT-qPCR. Full-length CVd-V sequences available in GenBank were aligned with Vector NTI Advance 11.5 software (Invitrogen, Carlsbad, CA, USA). The generated consensus sequence was used as a reference to design the primers and the TaqMan probe. The primer and probe position were based on the CVd-V sequence Acc. No. AB560862. The probe was designed within the amplicon region and labelled at the 5′ end with the reporter dye FAM (6-carboxyfluorescein) and at the 3′ end with a black hole quencher 1 (BHQ1). The Nucleotide-BLAST algorithm (https://blast.ncbi.nlm.nih.gov/Blast.cgi, accessed on 5 February 2025) was utilized to detect possible hybridization of the primers and probe with non-target organisms. Predicted secondary structures and hairpins were checked with the Oligo Analyzer Tool (https://eu.idtdna.com/calc/analyzer, accessed on 5 February 2025). In silico hybridization analyses against the complete genomes of all known citrus-infecting viroids (HSVd, CEVd, CDVd, CBLVd, CBCVd, CVd-VI, and CVd-VII) were performed using Vector NTI.

### 2.2. RT-qPCR Conditions

The RT-qPCR assay was performed in a reaction mixture of 20 µL final volume, containing 3 µL of total RNA, 0.5 µM of forward and reverse primers, 0.25 µM of TaqMan probe, 1× CAPITAL™ 1-Step qRT-PCR Probe Master Mix (Biotechrabbit GmbH, Berlin, Germany), 1 µL of RTase with RNase Inhibitor (Biotechrabbit GmbH, Berlin, Germany), and H_2_O DEPC water to reach the final volume. A Rotor-Gene Q2plex HRM Platform Thermal Cycler (Qiagen, Hilden, Germany) was used to perform the assay, with the following cycling conditions: reverse transcription at 50 °C for 10 min, enzyme denaturation at 95 °C for 10 min, and 45 cycles of 95 °C for 15 s and 60 °C for 60 s, with fluorescence measured at the end of each cycle. Three technical replicates were carried out.

### 2.3. Specificity of the RT-qPCR

To test the RT-qPCR assay specificity, total RNA extracts stored at the SAAF department of the University of Palermo and previously obtained from two citrus samples infected with CVd-V and from seven citrus samples, each solely infected with HSVd, CEVd, CDVd, CBLVd, CBCVd, CVd-VI, and CVd-VII, respectively, were analyzed. Each sample was tested in duplicate, and the negative controls from a healthy citrus plant and a no-template control were included.

### 2.4. Standard Plasmid Preparation

Standard plasmids for RT-qPCR sensitivity testing were prepared as follows: the complete RNA genome of CVd-V (isolate DW18, GenBank Accession no. PV657136) [18] was amplified and purified as described in Bani Hashemian and co-workers [24]. The purified products were inserted into a pGEM-T cloning vector (Promega, Madison, WI, USA) following the manufacturer’s instructions and subsequently transformed into One Shot™ Mach1™ competent *Escherichia coli* cells (Invitrogen Ltd., Paisley, UK). The transformant cells were subjected to ampicillin resistance selection, and a colony-PCR was performed to assess the insert presence. Plasmid DNAs were subsequently purified using the GenUP^®^ Plasmid kit (Biotechrabbit GmbH, Berlin, Germany) and quantified twice using a NanoDrop 1000 spectrophotometer (Thermo Fisher Scientific, Waltham, MA, USA). The plasmids were then sequenced in both directions by Macrogen Europe (Amsterdam, The Netherlands), and the obtained sequences were confirmed using the BLAST algorithm available at the NCBI website. One plasmid was selected for subsequent experiments (named pCVD-V#01). The plasmid copy number was calculated using the following formula:

$$No.\;of\;copies=\frac{DNA\;amount\left[ng\right]\times 6.022\times \;10^{23}}{DNA\;template\;lenght[bp]\times 1\times 10^{9}\times 650}$$

### 2.5. Standard Curve and Analytical Sensitivity

To generate a standard curve and assess the limit of detection (LoD) of the qPCR assay in controlled conditions, a 10-fold serial dilution of the pCVD-V#01 plasmid, starting from 40 ng/μL to 40 × 10^−10^ ng/μL, was used. The amplification was performed with three replicates, following the protocol described in Section 2.2, using 1 µL of recombinant plasmid. Molecular-grade water was used as the no-template control (NTC). The standard curve was obtained by plotting the threshold cycle (Ct) values against the logarithm of the plasmid dilutions. The amplification efficiency was calculated from the slope of the corresponding curve using the formula 10^(−1/slope)^.

### 2.6. Comparison Between CVd-V qPCR and Conventional PCR

The analytical sensitivity of the developed qPCR assay was subsequently compared with a conventional end-point PCR, using the plasmid serial dilutions previously obtained. The end-point PCR assay was performed in a 25 µL final reaction volume, containing 1 µL of diluted recombinant plasmid, 0.4 mM dNTPs, 2 mM MgCl_2_, 1 µM of each primer [24], 1× PCR Reaction Buffer (Biotechrabbit GmbH, Berlin, Germany), 2U of Taq DNA polymerase (Biotechrabbit GmbH, Berlin, Germany), and molecular-grade water to reach the final volume. The reaction was carried out in a MultiGene OptiMax thermal cycler (Labnet International Inc., Edison, NJ, USA) using the following cycling conditions: 95 °C for 5 min, 45 cycles of 30 s at 95 °C, 30 s at 59 °C, 30 s at 72 °C, and a final extension of 10 min at 72 °C. The PCR products were electrophoresed on 1.5% (*w*/*v*) agarose gel and stained with SYBR Safe (Thermo Fisher Scientific, Waltham, MA, USA).

### 2.7. Plant Material Collection

In order to evaluate the CVd-V dispersion in Sicily, a survey was conducted in commercial orchards across the main citrus-producing areas (Catania, Messina, and Siracusa provinces), as well as in a germplasm collection containing old varieties introduced from foreign countries that are no longer (or have never been) commercialized. The list of collected samples is reported in Supplementary Table S1. Each sample consisted of 10 leaves per plant, randomly collected during spring 2025. A total of 111 samples were collected from asymptomatic plants (80 samples from commercial orchards, 31 samples from germplasm collection) and stored at 4 °C before molecular analyses.

### 2.8. Total RNA and Crude Sample Extraction

Total RNA of the 111 samples collected was extracted using the GenUP^®^ Plant RNA kit (Biotechrabbit GmbH, Berlin, Germany), following the manufacturer’s instructions, with minor modifications. In detail, the petioles and the midvein of all the collected citrus leaves were homogenized in an extraction bag using the HOMEX 6 homogenizer (Bioreba, Reinach, Switzerland), with 6 mL extraction buffer (1.3 g sodium sulphite anhydrous, 20 g polyvinylpyrrolidone MW 24–40.000, 2 g albumin chicken egg grade II, 0.5% Tween-20, all dissolved in one L of distilled water at pH 7.4) [25]. Subsequently, 450 µL of each sample extract was added to 400 µL of the lysis buffer supplied with the kit, and the manufacturer’s protocol was followed from this step. The total RNA concentration of each extracted sample was measured twice using a NanoDrop 1000 spectrophotometer (Thermo Fisher Scientific, Waltham, MA, USA), subsequently adjusted to a final concentration of ≈50 ng/µL, and finally stored at −20 °C.

A quick and low-cost sample preparation method, named Petiole-Disc Crude Extract (PDCE), developed by Panno and coworkers [26], with minor corrections, was also used to compare its performance with that of conventional total RNA extracts. In detail, ten fresh-cut petioles from each sample collected were impressed onto 1 cm^2^ of Hybond-N+ hybridization membrane (GE Healthcare, Chicago, IL, USA), dried at room temperature for 5 min, and then placed in a 1.5 mL tube containing 0.1 mL of glycine buffer (0.1 M glycine, 0.05 M NaCl, 1 mM EDTA). The tubes were vortexed for 30 s and incubated at 95 °C for 10 min. Subsequently, 3 μL of the crude extract were utilized for the following steps.

### 2.9. Total RNA and Crude Plant Extract Comparison Using RT-qPCR and Sensitivity Evaluation Between RT-qPCR and Conventional RT-PCR

To analyze the 111 samples collected, the RT-qPCR developed in this work was carried out using both total RNA and Petiole-Disc Crude Extracts (PDCE). All tests included a healthy citrus total RNA as a negative control (NC) and molecular-grade water as NTC.

Total RNA extracts were also analyzed through two-step end-point RT-PCR [24]. In detail, the reverse-transcription step of the RT-PCR was performed in a final reaction volume of 20 µL, containing 0.4 mM dNTPs, 1× SSIV Buffer (Thermo Fisher Scientific, Waltham, MA, USA), 5 mM DTT, 1 µM of CVd-V specific reverse primer [24], 20 U of SuperScript IV reverse transcriptase (Thermo Fisher Scientific, Waltham, MA, USA), 3 µL of total RNA, and RNase-free water up to the final volume. The conditions for the retro-transcription were as follows: 65 °C for 5 min, 53 °C for 10 min, and 80 °C for 10 min. The PCR amplification step was subsequently carried out using 2 µL of RT product, as described in Section 2.6.

## 3. Results

### 3.1. RT-qPCR Primers and Probe Design and Specificity of CVd-V RT-qPCR

A set of two primers and a TaqMan probe named CVd-VF273, CVd-VR68, and CVd-VP18 (Table 1) were obtained within a conserved 105 bp region of the CVd-V genome to set up an RT-qPCR assay. In silico analysis performed using BLASTn revealed no cross-reactions with non-target organisms, and no secondary structures and hairpins were revealed using the Oligo Analyzer Tool. In addition, hybridization analysis performed with Vector NTI 11.5 confirmed the absence of significant homology with other citrus-infecting viroids.

To test the specificity of the RT-qPCR assay, total RNA extracted from citrus samples naturally infected with the seven other citrus-infecting viroids were used as outgroups and compared with total RNA from two CVd-V positive control samples. While the CVd-V positive samples produced a clear fluorescent signal, none of the other outgroups tested was detected, nor were the negative controls (Table 2), indicating the absence of cross-reactions.

### 3.2. qPCR Assay Analytical Sensitivity and Comparison with End-Point PCR

**Figure 1:**
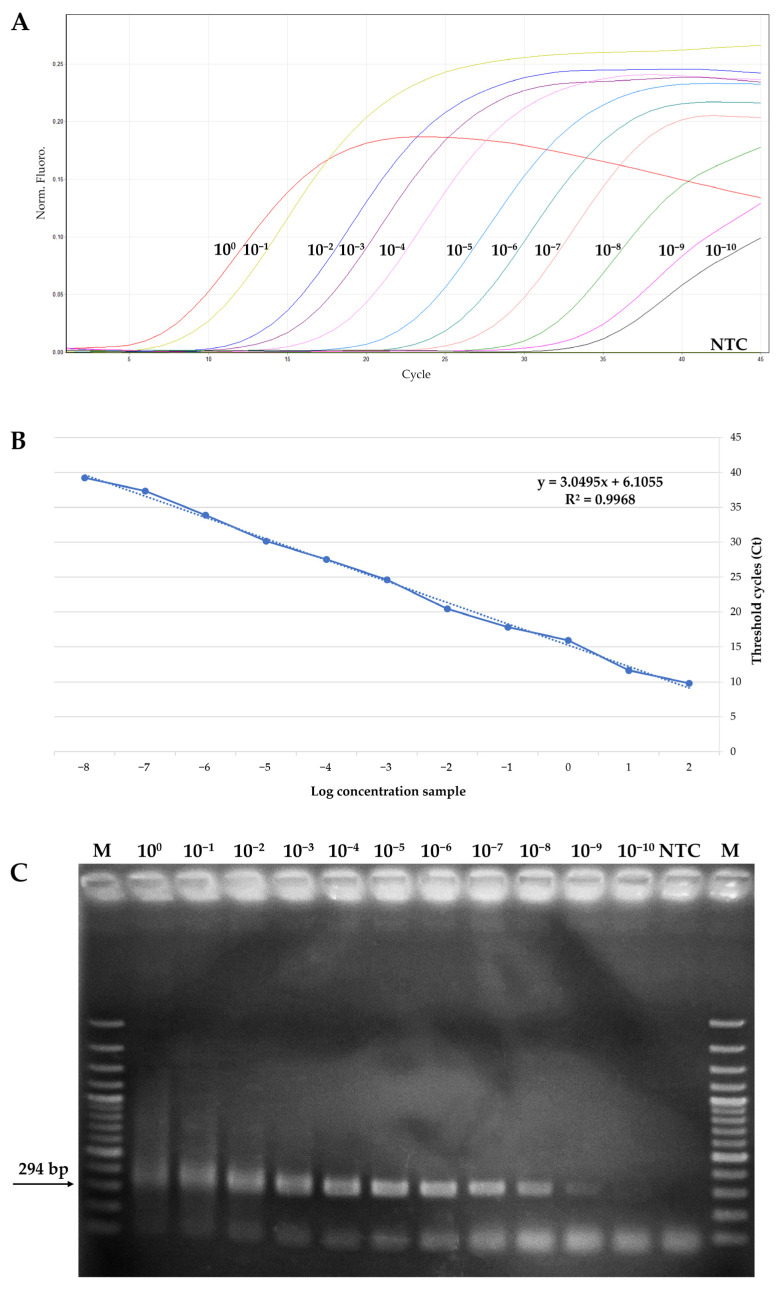
Ten-fold serial dilutions of purified recombinant plasmid. (**A**) Representative amplification plot showing qPCR fluorescence detection of the 10-fold serial dilutions, from 10^0^ (40 ng/μL) to 10^−10^ (40 × 10^−10^ ng/μL). (**B**) Linear regression analysis obtained by plotting the Ct values on the *Y*-axis against the logarithm of the initial plasmid copy number on the *X*-axis. Each data point represents the mean Ct value calculated from three technical replicates. R^2^: calculated correlation coefficient value. (**C**) Agarose gel electrophoresis (1.5%) of end-point PCR products obtained using the same serial dilutions (M: GeneRuler 100 bp plus DNA ladder; NTC: no-template control).

A standard curve was generated to calculate the analytical sensitivity threshold of the CVd-V qPCR assay using 10-fold serial dilutions of pCVDV#01 plasmid, ranging from 40 ng/µL (1.123 × 10^10^ copies) to 40 × 10^−10^ ng/µL (1.123 copies). The assay yielded a positive detection signal for all the dilutions tested (SD ± 0.2), with a minimum detectable copy number of 1.123 copies. No amplification was detected for the no-template control, even at late cycle thresholds (Figure 1A). The resulting standard curve exhibited a strong linear relationship, with a correlation coefficient of 0.9996 (Figure 1B). The same plasmid dilutions were also analyzed by conventional end-point PCR to compare the analytical sensitivity of the two techniques. End-point PCR was able to reliably detect clear bands generated by CVd-V amplification only up to 40 × 10^−9^ ng/µL (1.123 × 10^2^ copies) (Figure 1C), and although a faint band could appear visible at the 10^−10^ dilution, it was not reproducibly detected and was therefore considered below the reliable detection threshold. This indicates that the RT-qPCR assay was approximately 10-fold more sensitive.

### 3.3. Total RNA and Crude Plant Extract Comparison Using RT-qPCR and Sensitivity Evaluation Between RT-qPCR and Conventional RT-PCR

The 111 collected samples were analyzed by RT-qPCR using either total RNA or PDCE as templates, and also by end-point RT-PCR using total RNA, to compare the two assays’ performance in real plant matrices. All samples that tested positive by RT-qPCR using total RNA also showed positive signals when PDCE was used, although with higher Ct values. In contrast, CVd-V was not detected by conventional end-point PCR in several total RNA samples. All samples that tested negative by RT-qPCR also showed no amplification by end-point RT-PCR or when PDCE was used as template. In Table 3 are reported all samples that resulted positive for CVd-V infection.

RT-qPCR analysis detected CVd-V in 9 out of 111 total citrus samples, corresponding to an overall incidence of 8.1%. Among the positive samples, 4 out of 31 plants from the germplasm collection tested positive (12.9%), whereas the remaining 5 positives were detected among 80 samples collected from commercial orchards (6.25%). All infected plants consisted of newly established sweet orange clones grafted onto citrumelo swingle or sour orange rootstocks, except for one lemon grafted onto citrumelo swingle, and were asymptomatic, confirming that different citrus hosts can harbor CVd-V without showing visible symptoms.

## 4. Discussion

Citrus trees are constantly threatened by viral and viral-like diseases. Viroidal infections risk has increased in the Mediterranean basin [27], especially following regulatory restrictions on the use of sour orange rootstocks, and their replacement with CTV-tolerant rootstocks that are susceptible to viroids [7,8]. The management of these pathogens is challenging because they spread through infected propagation material [28], and infected plants often remain asymptomatic for several years after inoculation. Furthermore, multiple viroids often coexist in the same plant [20,29], causing synergistic interactions that exacerbate symptom severity [21], resulting in significant yield losses and economic damage. Additionally, viroid detection has always been a challenging task due to the limited availability of sensitive and rapid diagnostic tools, which restricts routine orchard surveillance and contributes to the underestimation of viroid incidence.

In this study, we developed a sensitive and specific RT-qPCR assay for CVd-V detection, a pathogen recently reported in Italy for the first time [18], and potentially capable of causing severe damage to citrus orchards when present alone or in mixed infection with other viroids. To assess the incidence and spread of CVd-V in Sicily, citrus samples from different Sicilian provinces, species, and grafting combinations were collected. The RT-qPCR assay proved to be robust and reliable for CVd-V detection when combined with total RNA extraction.

Analytical sensitivity analysis using recombinant plasmid standards showed that the qPCR was approximately 10-fold more sensitive than conventional end-point PCR assay in controlled conditions, with a detection limit of 1.123 copies (40 × 10^−10^ ng/µL), being in the range of the detection limit described for other viroids [30,31,32]. Additionally, the molecular technique for CVd-V detection proposed in this work showed similar sensitivity compared with other assays reported in the literature. In fact, Chambers and coworkers [33] developed a multiplex TaqMan RT-qPCR for the simultaneous detection of citrus viroids, including CVd-V, using only one pair of primers and different species-specific probes. However, this type of multiplex assay might yield false negative results when multiple viroids are present in the sample at different titers, due to the competition for primers, making the RT-qPCR described in the current work more reliable for CVd-V detection. Another real-time assay for CVd-V detection, consisting of two sets of degenerate primer pairs for the detection of all citrus viroids, was patented [34], but does not have the same sensitivity and specificity, due to the use of SYBR Green RT-qPCR technology. Compared to this assay, the technique developed in the present study again appears to have higher sensitivity, in addition to being specific only for CVd-V.

Field sample screening revealed that 9 out of 111 samples were positive for CVd-V. Among these, the 5 that tested positive were part of 80 samples collected from commercial orchards, resulting in an incidence of 6.25%, with infections detected in one Lunario VCR lemon and in Tarocco and Sanguinello sweet orange, two historically important blood orange cultivars selected in the early 1900s. It is likely that CVd-V has been present since that period but remained undetected due to limitations of earlier diagnostic approaches. Moreover, biological indexing on Etrog citron is not fully specific for CVd-V detection.

Additionally, to assess the assay performance in comparison with another common detection method for CVd-V, the RNA extracted from field samples was also analyzed by RT-PCR, and the obtained results confirmed that the RT-qPCR developed in this study can detect CVd-V more efficiently than the conventional RT-PCR. Furthermore, a rapid and cost-effective sample preparation method, consisting of impressing fresh-cut citrus petioles on a positively charged hybridization nylon membrane [35,36], was also evaluated to assess its suitability for the CVd-V RT-qPCR assay. This approach successfully detected CVd-V, albeit with higher Ct values, indicating reduced sensitivity compared with total RNA extraction. Similar results were reported by Papayannis and coworkers [37], suggesting that crude extraction methods are generally suitable for viroid detection. This strategy opens the possibility to perform viroid detection directly in the field, using portable thermal cyclers and ready-to-use molecular diagnostic kits [25,38], significantly accelerating routine surveillance of citrus orchards.

Overall, although data on CVd-V distribution remain limited, the low incidence observed in this study is comparable to reports from other countries. Infection rates of 4.7% (42 samples analyzed) in China [13], 25% (20 samples analyzed) in Turkey [39], 36.8% (38 samples analyzed) in Tunisia [17], and 24.3% (152 samples analyzed) in Japan [16] have been reported, whereas much higher rates (up to 93%) were observed in Pakistan [40]. Given the sporadic nature of surveys conducted over time, it is likely that CVd-V is more widespread than currently recognized.

## 5. Conclusions

In conclusion, the RT-qPCR assay developed in this study showed excellent diagnostic sensitivity and specificity, representing a valuable tool for routine screening of commercial orchards and propagation material. Furthermore, this study provided the first report on the incidence of CVd-V in Sicily and highlights the need for broader surveys across the Italian citrus-growing regions.

## Figures and Tables

**Table 1:** CVd-V specific primers and probes.

Name	Genomic Position *	Sequence 5′-3′	Amplicon Size (bp)
CVd-VF273	273	CACGATTGGTGTTCTCCCTGG	105
CVd-VP18	18	FAM–GGTTCCTGTGGGTCACCCCGCCC–BHQ1	
CVd-VR68	68	CGACGACAGGTGAGTACTCT	

* Based on the CVd-V sequence Acc. No. AB560862.

**Table 2:** Specificity of CVd-V RT-qPCR assay against different citrus-infecting viroids.

ID Sample	Ct Value
CVd-V-01	26.55 ± 0.2
CVd-V-02	24.26 ± 0.3
HSVd	-
CEVd	-
CDVd	-
CBCVd	-
CBLVd	-
CVd-VI	-
CVd-VII	-
pCVD-V#01 [40 ng/µL]	7.16 ± 0.2
Healthy citrus RNA (NC)	-
Molecular-grade water (NTC)	-

**Table 3:** CVd-V positive samples detected by comparing total RNA and the Petiole-Disc Crude Extracts analyzed by RT-qPCR, and total RNA by end-point RT-PCR.

ID Sample	Scion/Rootstock	RT-qPCR Ct Value (Total RNA)	RT-qPCR Ct Value (PDCE)	RT-PCR (Total RNA) *
Imp18	“Sanguinello” sweet orange/Sour orange	23.02	28.11	+
Imp18.2	“Tarocco rosso” sweet orange/Sour orange	24.26	31.56	+
Imp18.3	“Tarocco rosso” sweet orange/Sour orange	23.64	29.46	+
B-F8 P5	“Femminello Continella” lemon/Citrumelo swingle	35.11	39.31	-
41A	“Moro nuc. 58-8d-1” sweet orange/Citrumelo swingle	38.80	39.96	-
42A	“Tarocco rosso VCR” sweet orange/Citrumelo swingle	34.71	39.08	+
43B	“Navelina VCR” sweet orange/Citrumelo swingle	35.88	39.38	-
17A	“Tarocco Meli nuc. C8158” sweet orange/Citrumelo swingle	35.82	38	+
44A	“Lunario VCR” lemon/Citrumelo swingle	35.81	40.92	-

* (+): positive sample; (-): negative sample.

## Data Availability

The original data presented in the study are openly available in Supplementary Table S1.

## Supplementary Materials

The following supporting information can be downloaded at: https://www.mdpi.com/article/10.3390/v18030335/s1, Table S1: hosts, varieties, grafting combination and collection site of the samples analyzed.
