# Supplementary material for: Development of a Rapid and Sensitive RT-qPCR for the Specific Detection of Citrus Viroid V and Its Field Application
Source: Viruses. 2026 Mar 9;18(3):335. doi: 10.3390/v18030335 (PMC13030860; doi:10.3390/v18030335)
Supplement: Supplementary file 1 [file viruses-18-00335-s001.zip › viruses-4166516-supplementary.pdf]

Supplementary Table S1 – Hosts, varieties, grafting combination and collection site of the samples collected

ID sample	Host	Variety	Rootstock	Province collection	Field	Germplasm
Atl 39	Atalantia	Ceylanica	Citrange troyer	Messina	X	
Atl P7	Atalantia	Ceylanica	Citrange troyer	Messina	X	
Atl 0	Atalantia	Ceylanica	Citrange troyer	Messina	X	
3A	Bergamot	Fantastico VCR	Citrumelo Swingle	Catania	X	
11A	Bergamot	Fantastico VCR	Citrumelo Swingle	Siracusa	X	
B-F28 P11	Citron	Vozza Vozza	Citrumelo Swingle	Catania	X	
30A	Citron	Diamante VCR	Citrumelo Swingle	Catania		X
B-F20 P5	Clementine	Nova	Citrumelo Swingle	Catania	X	
B-F25 P5	Clementine	Romano	Citrumelo Swingle	Catania	X	
B-F25 P6	Clementine	Nour	Citrumelo Swingle	Catania	X	
18A	Clementine	Caffin	Citrumelo Swingle	Catania		X
21A	Clementine	Spinoso VCR	Citrumelo Swingle	Catania		X
28A	Clementine	Hernandina VCR	Citrumelo Swingle	Catania		X
2A	Clementine	Corsica 2	Citrumelo Swingle	Catania		X
4A	Clementine	Comune ISA VCR	Citrumelo Swingle	Catania	X	
Nova Gent.	Clementine	Nova	Citrange	Siracusa	X	
12A	Clementine	SRA 89	Citrumelo Swingle	Siracusa	X	
800A/BIS	Fortunella	Obovata	Citrumelo Swingle	Catania	X	
Asgv3	Fortunella	Obovata	Citrumelo Swingle	Catania	X	
Nova CT GA	Fortunella	Obovata	Citrumelo Swingle	Siracusa	X	
B- F27 P7	Grapefruit	Oroblanco	Sour orange	Catania	X	
32A	Grapefruit	Star ruby	Citrumelo Swingle	Catania		X
Oroblanco ABT	Grapefruit	Oroblanco	Sour orange	Catania	X	
B-F27 P8	Hybrid	Citradia	Citrumelo Swingle	Catania	X	
B-F30 P13	Hybrid	Siamelo	Citrumelo Swingle	Catania	X	
Sl19.2	Hybrid	Tacle	Sour orange	Catania	X	
B-F11 P2	Lemon	Kamarina	Sour orange	Catania	X	
B-F23 P13	Lemon	Incomparabile	Sour orange	Catania	X	
B-F24 P13	Lemon	Doppio	Citrumelo Swingle	Catania	X	
B-F25 P13	Lemon	Doppio Lentini	Sour orange	Catania	X	
B-F26 P8	Lemon	Limone del Borneo	Citrumelo Swingle	Catania	X	
B-F5 P1	Lemon	Akragas	Citrumelo Swingle	Catania	X	
B-F8 P1	Lemon	Segesta	Sour orange	Catania	X	
B-F8 P5	Lemon	Femminello Continella	Citrumelo Swingle	Catania		X
19A	Lemon	Interdonato	Citrumelo Swingle	Catania		X
1A	Lemon	Zagara bianca Nuc. 356	Citrumelo Swingle	Catania		X
25A	Lemon	Verna	Citrumelo Swingle	Catania		X
31A	Lemon	Fino 49	Citrumelo Swingle	Catania		X
37A	Lemon	Fino 95	Citrumelo Swingle	Catania		X
40B	Lemon	Sfusato amalfitano IAM-UA A0008	Citrumelo Swingle	Catania	X	
44A	Lemon	Lunario VCR	Citrumelo Swingle	Messina	X	
6A	Lemon	Sfusato amalfitano IAM-UA A0008	Citrumelo Swingle	Messina	X	
9A	Lemon	Femminello Siracusano 2KR	Citrumelo Swingle	Messina	X	
Ma 1 Yellow	Lemon	Femminello	Sour orange	Messina	X	
Mirmi1	Lemon	Femminello	Macrophylla	Siracusa	X	
B-F27 P11	Lime	Pursha × Chinotto	Citrumelo Swingle	Catania	X	
23A	Mandarin	Avana apireno Nuc. 62-ap9	Citrumelo Swingle	Catania		X
34B	Mandarin	Tardivo di ciaculli Nuc. 60-22a-7	Citrumelo Swingle	Catania		X
38B	Mandarin	Avana apireno Nuc. 62-ap9	Citrumelo Swingle	Catania	X	
7A	Mandarin	Tardivo di Ciaculli Nuc. 60-22a-7	Citrumelo Swingle	Messina	X	
Primosole2	Mandarin-like hybrid	Primosole	Volkameriana	Messina	X	
Primosole3	Mandarin-like hybrid	Primosole	Volkameriana	Messina	X	
Primosole8	Mandarin-like hybrid	Primosole	Volkameriana	Messina	X	
B-F28 P14	Pummelo	Pummelo locale	Sour orange	Catania	X	
Aa8	Sour orange	Sour orange	-	Messina	X	
800A	Sweet orange	Valencia	Citrumelo Swingle	Catania	X	
Collinetta 1	Sweet orange	Tarocco Sciara	Citrumelo Swingle	Catania		X

Imp 18.1	Sweet orange	Tarocco rosso	Sour orange	Catania		X
14B	Sweet orange	Navelina VCR	Citrumelo Swingle	Catania		X
15A	Sweet orange	Nour	Citrumelo Swingle	Catania		X
16A	Sweet orange	Tarocco TDV	Citrumelo Swingle	Catania		X
17A	Sweet orange	Tarocco Meli Nuc. C8158	Citrumelo Swingle	Catania		X
20A	Sweet orange	Tarocco Gallo VCR	Citrumelo Swingle	Catania		X
22A	Sweet orange	Sanguinello moscato Nuc. 49-5-5	Citrumelo Swingle	Catania		X
24A	Sweet orange	Fukumoto	Citrumelo Swingle	Catania		X
26A	Sweet orange	Tarocco Lempso	Citrumelo Swingle	Catania		X
27A	Sweet orange	Tarocco Sant'Alfio	Citrumelo Swingle	Catania		X
29A	Sweet orange	Newhall VCR	Citrumelo Swingle	Catania		X
33B	Sweet orange	Tarocco Lempso	Citrumelo Swingle	Catania		X
35B	Sweet orange	Tarocco Ippolito m 507	Citrumelo Swingle	Catania		X
36B	Sweet orange	Lane late INIASEL 198	Citrumelo Swingle	Catania		X
39A	Sweet orange	Navel cara cara	Citrumelo Swingle	Catania	X	
41A	Sweet orange	Moro Nuc. 58-8d-1	Citrumelo Swingle	Catania		X
42A	Sweet orange	Tarocco rosso VCR	Citrumelo Swingle	Catania		X
43B	Sweet orange	Navelina VCR	Citrumelo Swingle	Catania	X	
5A	Sweet orange	Lane late INIASEL 198	Citrumelo Swingle	Catania	X	
CP13	Sweet orange	Hamlin	Sour orange	Catania	X	
Sl10.2	Sweet orange	Navelina	Sour orange	Catania	X	
B282	Sweet orange	Tarocco TDV	Citrange Troyer	Catania	X	
B497	Sweet orange	Tarocco Tapi	Citrange Troyer	Catania	X	
Sl1.1	Sweet orange	Vaniglia	Sour orange	Catania	X	
Canneto3	Sweet orange	Tarocco Scirè VCR	Sour orange	Catania	X	
Imp18	Sweet orange	Sanguinello	Sour orange	Catania	X	
LCut 1	Sweet orange	Moro	Citrange Troyer	Catania	X	
LCut 2	Sweet orange	Moro	Citrange Troyer	Catania	X	
Vaniglia VGL	Sweet orange	Vaniglia	Sour orange	Catania	X	
8A	Sweet orange	Tarocco Ippolito M507	Citrumelo Swingle	Catania	X	
Moro2	Sweet orange	Moro	Sour orange	Catania	X	
Motta X	Sweet orange	Tarocco rosso	Citrange Carrizo	Catania	X	
H/Aa7	Sweet orange	Hamlin	Sour orange	Messina	X	
H/Aa9	Sweet orange	Hamlin	Sour orange	Messina	X	
Hu3.1	Sweet orange	Hamlin	Sour orange	Messina	X	
Hu3.2	Sweet orange	Hamlin	Sour orange	Messina	X	
Imp18.2	Sweet orange	Tarocco rosso	Sour orange	Siracusa	X	
Imp18.3	Sweet orange	Tarocco rosso	Sour orange	Siracusa	X	
Sl4.2	Sweet orange	Tarocco Gallo	Sour orange	Siracusa	X	
Sl6.1	Sweet orange	Tarocco Gallo	Sour orange	Siracusa	X	
Lf 025	Sweet orange	Tarocco Ippolito	Sour orange	Siracusa	X	
Pine7	Sweet orange	Pineapple	Macrophylla	Siracusa	X	
Bov	Sweet orange	Navelina	Sour orange	Siracusa	X	
P1R1	Sweet orange	Tarocco	Sour orange	Siracusa	X	
10A	Sweet orange	Washington Navel CRC 3033	Citrumelo Swingle	Siracusa	X	
13A	Sweet orange	Navelina VCR	Citrumelo Swingle	Siracusa	X	
Sl16.2	Sweet orange	Tarocco Sciara	Sour orange	Siracusa	X	
Quali10	Sweet orange	Lempso	Macrophylla	Siracusa	X	
Sl15.2	Sweet orange	Tarocco Sciara	Sour orange	Siracusa	X	
SPG1	Sweet orange	Moro	Sour orange	Siracusa	X	
SPG2	Sweet orange	Moro	Sour orange	Siracusa	X	
SPG3	Sweet orange	Moro	Forner Alcaide	Siracusa	X	
SPG4	Sweet orange	Moro	Forner Alcaide	Siracusa	X	
TO	Tangelo	Orlando	Macrophylla	Catania	X	
